# ASO Author Reflections: Dilemma of Paraaortic Lymph Node Metastases During Exploration for Suspected Periampullary Carcinoma

**DOI:** 10.1245/s10434-020-08348-2

**Published:** 2020-03-18

**Authors:** Bobby K. Pranger, Vincent E. de Meijer

**Affiliations:** Department of Hepatopancreatobiliary Surgery and Liver Transplantation, University Medical Center Groningen, University of Groningen, Groningen, The Netherlands

## Past

The decision to perform a pancreatoduodenectomy for suspected periampullary cancer depends on vascular involvement, distant metastases, and lymph node metastases. Lymph node involvement of the paraaortic lymph node (PALN; station 16) corresponds with distant metastases according to the Japanese Pancreas Society Classification of Pancreatic Cancer but not in the current edition of the AJCC Cancer Staging Manual. Also, the International Study Group on Pancreatic Surgery does not include PALN in standard lymphadenectomy for pancreatoduodenectomy.^1^ Poor survival after pancreatoduodenectomy with positive PALN has been seen previously, but its prognostic significance has not been established.^2^ Some studies have identified patients who might benefit from resection, while others routinely perform a palliative bypass procedure when positive PALNs are found during exploration.^3^ The optimal strategy to approach PALN during surgery remains unclear.

## Present

This study compared the outcome of patients with suspected periampullary cancer in whom PALNs were routinely sampled during surgical exploration. Patients with PALN involvement who underwent resection had a median overall survival of 11 (95% CI 8.8–13.2) months, compared with 7 (95% CI 5.5–8.5) months in patients who received a palliative bypass (*P* = 0.049). The difference remained 2 months in a subgroup of patients who received adjuvant chemotherapy (*P* = 0.033). The survival benefit in patients who underwent pancreatoduodenectomy came at the cost of increased morbidity. Given that severe comorbidity (ASA grade ≥ 3) was associated with decreased survival, we concluded that a pancreatoduodenectomy could be considered in selected fit patients with PALN metastases because it offers survival benefit, albeit at the risk of increased morbidity.^4^

## Future

Virtually all studies on the prognostic role of PALN have followed a retrospective study design. Currently, an ongoing prospective cohort study led by the Dutch Pancreatic Cancer Group is investigating the prognostic impact of lymph node metastases at the common hepatic artery (station 8), celiac trunk (station 9), and PALN on overall survival in patients undergoing pancreatoduodenectomy for suspected periampullary cancer.^5^ This study might further increase our understanding of the prognostic value of PALN metastases on overall survival after pancreatoduodenectomy for periampullary cancer.
